# Artemisia plants, arachidonic and other polyunsaturated fatty acids

**Published:** 2020-06-01

**Authors:** Jérôme Munyangi, Pierre Lutgen

**Affiliations:** 1 Faculté de Médecine,Université de Kolwezi, Democratic Republic of the Congo;; 2 IFBV-BELHERB, Luxembourg

## Abstract

Arachidonic acid (AA or ARA) is an extremely important fatty acid involved in cell regulation. It is a polyunsaturated fatty acid (20:4n6) covalently bound in esterified form in membrane phospholipids of most body cells. Following irritation or injury, arachidonic acid is released and oxygenated by enzyme systems leading to the formation of an important group of inflammatory mediators, to the prostaglandins (PGE₂) by the cyclooxygenase enzyme. This paper describes the positive health effects of arachidonic acid on malaria and other tropical diseases.

## Introduction

Over recent decades polyunsaturated fatty acids (PUFA), especially ω-3 (omega-3) and ω-6 (omega-6), have acquired fame in the health business, which they may deserve or not.

Arachidonic acid is a 20-carbon ω-6 conditionally essential fatty acid. It sits at the head of the "arachidonic acid cascade" – more than 20 different signaling paths that control a wide array of bodily functions, but especially those functions involving inflammation, cell growth and the central nervous system. Most AA in the human body derives from dietary linoleic acid (another essential fatty acid, 18:2 ω-6), which is derived from nuts, seeds, vegetable oils and animal fats.

AA is present throughout the body and comprises the greatest % weight of total fatty acids among long chain poly-unsaturated fatty acids (LC-PUFAs). It is present as a structural component in animal and human cell membrane phospholipids, skeletal muscles, the brain, and liver.

Arachidonic acid is present in red meat, eggs, algae and fish oil. 0.1% in fatty meat, 0.7% in fish oil, 0.3% in eggs, and 0.4% of the total fat of breast milk.

In breast milk it is on average 0.5% by wt. The IUPAC Lipid Handbook confirms that human milk contains arachidonic acid, but cow milk much less [1-4].

It is especially necessary during periods of bodily growth or repair and is thus a natural and important component of breast milk [5].

Without the provision of preformed arachidonic acid in human milk the growing infant cannot maintain AA levels from the synthetic pathways alone that are sufficient to meet metabolic demand. During late infancy and early childhood, the amount of dietary arachidonic acid provided by solid foods is low.

In most mammals, linoleic acid is converted into arachidonic acid. Some mammals lack the ability to —or have a very limited capacity to—convert linoleic acid into arachidonic acid, making it an essential part of their diets. Since little or no arachidonic acid is found in common plants, such animals are obligate carnivores; the cat is a common example.

Arachidonic acid plays the role of messenger. Free molecules may be reintegrated into the membrane phospholipids, or bound to plasmatic albumin, or metabolized by P450 cytochromes [6].

The release and recapture of arachidonic acid from phospholipids (process called cycle of Land) is an essential process for normal human growth, health promotion, disease prevention and for the repair of damaged cells [7].

Arachidonic acid in its free state is an oil which is not soluble in water. However, at the normal physiological pH interconversion between non-ionic forms into ionic, salty forms is possible. Arachidonic acid is characterized by a high pKa, which plays an essential role in this interconversion, and consequently regulates the distribution and binding in the cells and tissues. It interacts with albumin. This protein significantly reduces the concentration of fatty acids in human plasma [8,9].

Inflammation is the immune system^’^s response to infection and injury and has been implicated in the pathogeneses of arthritis, cancer and stroke, as well as in neurodegenerative and cardiovascular disease. Inflammation is an intrinsically beneficial event that leads to removal of offending factors and restoration of tissue structure and physiological function. The acute phase of inflammation is characterized by the rapid influx of blood granulocytes, typically neutrophils, followed swiftly by monocytes that mature into inflammatory macrophages that subsequently proliferate and thereby affect the functions of resident tissue macrophages. This process causes the cardinal signs of acute inflammation: rubor (redness), calor (heat), tumor (swelling) and dolor (pain). Once the initiating noxious stimulus is removed via phagocytosis, the inflammatory reaction can decrease and resolve. During the resolution of inflammation, granulocytes are eliminated and macrophages and lymphocytes return to normal pre-inflammatory numbers and phenotypes. The usual outcome of the acute inflammatory program is successful resolution and repair of tissue damage, rather than persistence and dysfunction of the inflammatory response, which can lead to scarring and loss of organ function. It may be anticipated, therefore, that failure of acute inflammation to resolve may predispose to autoimmunity, chronic dysplastic inflammation and excessive tissue damage. Prostaglandins function on both the promotion and resolution of inflammation in a kind of homeostasis [10,11].

Prostaglandins play a key role in the generation of the inflammatory response. Their biosynthesis is significantly increased in inflamed tissue and they contribute to the development of the cardinal signs of acute inflammation. While the pro-inflammatory properties of individual prostaglandins during the acute inflammatory response are well established, their role in the resolution of inflammation is more controversial. But it is well known that arachidonic acid promotes skin wound healing [12].

PGE₂ have potent inflammatory properties and they are readily detectable in acute inflammatory exudates. Temperature is regulated in response to the hormone PGE₂. A modest fever may develop. Long regarded as a harmful by-product of infection, fever may instead be an ancient ally against disease. Redness, swelling and pain are normal primary effects of arachidonic acid. It ensures that our body responds properly to a physical insult or pathogen, and it also helps ensure that the inflammatory response is turned off when it^’^s no longer needed. It may momentarily exacerbate symptoms of joint pain [13].

It was already claimed in 1983 that PGE₂, derived from arachidonic acid, plays a clear role in the regulation of cellular and humoral responses [14].

PGE₂ regulates macrophage derived TNF-α. These studies confirmed that this prostaglandin can regulate macrophage activity and promote the phagocytosis of bacteria. Relatively little attention is paid to the observation that certain lipids could have antimicrobial actions and thus may serve as endogenous antibiotic-like actions. The importance of these antimicrobial lipids lies in the fact that they are present in all tissues of the body [15,16].

Studies in search of extracellular bactericidal factors for pneumococci revealed that the most anti-pneumococcal activity resided in the most highly unsaturated acid namely arachidonic acid (AA, 20:4n-6). Other unsaturated fatty acids: linoleic, oleic, and palmitoleic also showed anti-bacterial activity but were less potent compared to AA. AA was found to be active against gram-positive and gram-negative bacteria [17,18].

Several metabolites of arachidonic acid have potent effects on lung vascular and airway function. Some of these substances are released from the lungs when the lungs are diffusely injured [19].

The roles of arachidonic acid (AA) metabolites in hypoxia-induced pulmonary vasoconstriction, a critical physiological mechanism that prevents ventilation/perfusion mismatch, are still incompletely understood [20].

Indeed, caution needs to be taken concerning this effect of AA on pulmonary vasoconstriction. Linoleic acid may have immune-depressive effects and linoleic acid is a precursor of arachidonic acid in the human body. In-depth analysis of lipidomic profile in host cells infected with coronavirus has revealed that the LA to AA metabolism axis of those cells is the most perturbed lipidomic pathway with a concurrent increased production of AA due to both HCoV-229E and MERS-CoV infections. The same study showed that an exogenous supplementation of AA significantly reduced the replication of both strains indicating a potential disturbance on LA – AA axis by excess AA possibly through a feedback inhibition. Therefore, LA – AA axis is vital for the replication of this virus [21,22].

The toxicity and adverse effects of arachidonic acid have been assessed. The daily intake of a breastfed infant can be estimated at 140 mg. Children were given 400 mg daily for 15 days over 3 weeks, i.e. 5 days per week. Not a single child reported the slightest malaise or adverse reaction during, and six weeks after treatment. These results fully confirm the reports on safety of AA in athletes supplemented with 1,000 mg for 50 days.

## Polyunsaturated Fatty Acids and Arachidonic Acid Have Antimalarial Properties

AA and other PUFAs are cytotoxic to malaria parasites. PUFAs have been shown to have antimalarial effect. C18 fatty acids, such as oleic, elaidic, linoleic, and linoleic acids inhibited proliferation of malarial parasites in mice infected with *Plasmodium vinckei petteri* or with *P. yoelii nigeriensis*. *In vitro* studies revealed that C18 fatty acids can inhibit the growth of *P. falciparum*. The cytotoxic effect of the fatty acids is rather rapid and completely inhibited nucleic acids and protein syntheses in less than 30 min [23].

Many studies indicate the importance of cellular oxidative processes against parasite infections. These results are supported by other studies which showed that infections due to Leishmania, Trypanosoma and Schistosoma parasites can be treated successfully with PUFAs including AA both in experimental animals and humans [24,25].

But the efficiency of AA against *P. falciparum* is a complex issue as the parasite produces its own PGD2, PGE2, and PGF2a in a way that is clearly distinguishable from the PG biosynthesis by mammalian cyclooxygenase. However, it is important to notice that the capacity of the autogenous PG production was increased several-fold after cultivating the parasites with exogenously added arachidonic acid. Almost all of the parasite-produced PGs accumulated in the culture medium but not in the cell, because PGs could not be detected in the cell homogenate without incubation with exogenous AA. It was reported that arachidonic acid content in phospholipids of *P. falciparum*–infected RBC plasma membrane is much lower than that of normal RBC.

Why does *P. falciparum* produce PGs? The most intriguing possibility is that the parasite-produced PGs modulate the host defense mechanism against malaria infection. The addition of arachidonic acid however perturbs this homeostasis [26].

In a more recent study, it was shown that in comparison with oleic acid, eicosapentaenoic acid (EPA), docohexaeonic acid (DHA), only arachidonic acid enhanced PGE2, but reduced IL-10 IFNγ [27].

Already in 2000 it had been demonstrated in a study on Gabonese children with and without malaria that prostaglandins are important pro-inflammatory mediators of the host-immune response to infection [28].

The authors postulate that PGE₂ levels in healthy malaria-exposed children protects against malaria, while decreases in PGE₂ during acute malaria increase susceptibility to severe disease. This has been confirmed in Tanzanian children [29].

The authors suggest that high levels of PGE₂ in children with asymptomatic parasitemia may contribute to the maintenance of malaria tolerance, which is the ability to tolerate circulating parasites without fever. In children with cerebral malaria their production is impaired and this often leads to adverse outcomes.

Another hypothesis for the antimalarial properties of arachidonic acid is its influence on endothelial cell motion. Infected erythrocytes adhere to endothelial cells, especially in the case of severe malaria. If AA enhances endothelial cell motion, this will affect adhesion. One might even speculate that this has an influence on the motility of sporozoites in the blood vessels. Sporozoites are the marathon runners of the *Plasmodium* life cycle. They move in a stick-and-slip shuffle and if the blood vessel wall is slippery their movement will be inhibited. This could eventually explain the prophylactic efficacy of *Artemisia* infusions rich in AA [30].

Arachidonic acid inhibits G6PD generation and it is well known that G6PD deficiency protects against malaria. But G6PD deficiency may also open the door for hemolysis. Other long chain polyunsaturated fatty acids (PUFA) have a similar effect, saturated fatty acids don^’^t [31].

Arachidonic acid may also be converted into the hydroxyperoxy-HPETE-acid and attack *Plasmodium* like other peroxides do [32].

Unsaturated fatty acids strongly contribute to the neutrophil-mediated killing of *P. falciparum*. Saturated fatty acids have no effect. Unsaturated fatty acids with 18 carbon atoms also fail to enhance killing of the parasite. Optimum parasite killing is seen for 20:4 (n-6), 20:5 (n-3) and 22:6 (n-3) (AA, EPA, DHA). The neutrophil killing effect is further increased by the presence of the cytokine TNF-α. The hydroxy peroxides, especially HETE, of these fatty acids are very effective in the direct killing of the parasite and less by priming neutrophil-mediated killing. Neutrophils activated by PUFAs adhere to the parasite, leading to phagocytosis and killing [33].

This is one of the reasons why all *Plasmodium* parasites try to expel from infected erythrocytes as much arachidonic acid as possible but increases the concentration of low chain saturated fatty acids which they need.

Within human erythrocytes the rapid multiplication of the parasite also requires an active production of new membranes. It has been demonstrated that phosphatidylinositol (PI) and phosphatidylethanolamine (PC) phospholipids localize to the food vacuole membrane and the erythrocyte membrane. During invasion, this phospholipid is injected from the apical end of the merozoite into the host membrane. PC is the most abundant phospholipid in Plasmodium membranes followed by others like phosphatidylethanolamine (PE) or PS (phosphatidylserine).

The unsaturation index of PC is much lower than PE, and much lower in infected erythrocytes than in uninfected ones. Highly unsaturated fatty acids like arachidonic, docosahexaeonic are reduced by 70%, whilst palmitic and oleic acid are doubled [34]. This was already described in a report from WHO in 1977 [35], see table 1.

**Table 1:** Fatty acids of the total lipids of normal erythrocytes and of their malarial parasites.

Fatty acid	Duck erythrocytes	*Plasmodium lophurae*	Monkey erthrocytes	*Plasmodium knowlesi*	Rat erythrocytes	*Plasmodium berghei*
16:0	24	26	22	34	24	42
18:1	18	33	18	36	8	21
20:4	10	3	17	2	31	5
22:6	7	3	2	<1	-	-

The effect of arachidonic acid on bilharzia has been extensively studied at the University of Cairo [36,37].

Schistosomiasis trials were made with castor oil, praziquantel and fish oil. The oils, particularly castor oil, given by oral gavage for 7 days had the unexpected result to reduce cercarial penetration by 93% [38].

Trials have demonstrated that 5 mM arachidonic acid leads to irreversible killing of *ex vivo* 1-, 3-, 4-, 5- and 6- weeks old *Schistosoma mansoni* within 3 to 4 hours. This efficiency could be duplicated *in vivo* in a series of 6 independent experiments in mice. AA in pure form or included in an infant formula consistently led to a 40 to 80% decrease in total worm burden. Arachidonic acid is already marketed for human use in the United States and Canada. Royal DSM has introduced an international patent claiming prevention and treatment of schistosomiasis with arachidonic acid combined with praziquantel (WO/2015/123480).

Another clinical trial was run in Egypt on 66 schoolchildren comparing arachidonic acid with praziquantel. Arachidonic proved to be as efficacious as praziquantel: 78% and 85% cure rates respectively. Not a single child reported any adverse reactions during or after treatment with arachidonic acid. A majority of children treated with praziquantel reported headache, dizziness, abdominal pain, nausea and diarrhea [39,40].

Fish oil (rich in arachidonic acid) is effective against helminthic diseases like trichinellosis. A reduction of 30.6% of adult worms in Wistar rats was noticed in a fish oil group as compared to the standard diet group [41].

## Artemisia Plants and Fatty Acids

*Artemisia* plants, compared to other leafy vegetables, are rich in fatty acids. In the literature values of 3.31 – 17.78 mg/g are quoted for green Artemisia leaves, compared to 1,7 mg/g for spinach, 0.6 mg/ g for salad, 8.5 mg/g for parsley [42,43].

It is claimed that higher plants and vegetables do not produce or contain arachidonic acid. It is only found and extracted from mosses and algae. For this reason, several laboratories have tried to genetically modify plants for production of arachidonic acid. Patent US7943816 for example introduces foreign genes into soybeans to this effect. AA is an ancient metazoan signaling molecule, eliciting plant stress and defense signaling networks [44].

A phytochemical analysis of five Artemisia species in Turkey shows that saturated fatty acids in these plants represent on the average 40% of the total and the unsaturated fatty acids 60%, including those with antimalarial activities like linoleic acid, arachidonic acid and linolenic acid [45].

Very important even is the presence of eicosadieonic acid 20:2 n-6 EDA. 10% on the average in the five plants. EDA is generating prostaglandins [46].

Artemisia plants are rich in fungal endophytes with a great variety in species and it is likely that the build-up of arachidonic acid takes place there, as it is the case for *Mortierella alpina* [47-49].

In the laboratory arachidonic acid can be produced by the fungus *Mortierella alpina*, even at industrial scale [50-52].

## Artemisia and Platelet Activation

Activation of platelets appears to be a critical parameter in malaria. Activated platelets in malaria-infected patients are hypersensitive and enhance haemostatic responses [53,54].

Multiple pathways contribute to platelet activation including those dependent upon arachidonic acid. Arachidonic acid is released from the platelet membrane by phospholipase A2 action and is then metabolized in the cytosol by specific arachidonic acid oxidation enzymes including prostaglandin [55].

It would be important also to understand how *Artemisia* infusions play a role. *Artemisia* plant extracts increase and stabilize the platelet count in malaria infections and increase survival [56,57].

*Artemisia* infusions are also very efficient against diabetes as shown in a recent paper by us. Diabetes and obesity heavily contribute to the death toll related to the coronavirus. Maybe a similar lead should be investigated more in depth [58].

But this is not the case for artemisinin derivatives, which are strong pro-oxidants. Artesunate was shown to decrease hemoglobulin, erythrocyte and platelet count. Artemether caused significant reduction of the hematological profile of the animals in a dose dependent manner. This can probably aggravate anemia when artemether is administered to malaria patients [59,60].

## Conclusion

Arachidonic acid deserves more research in the fight against tropical and viral diseases.
